# Metal allergy patient treated by titanium implant denture: A case report with at least 4‐year follow‐up

**DOI:** 10.1002/ccr3.1753

**Published:** 2018-08-28

**Authors:** Huijiao Yan, Shaista Afroz, Junhel Dalanon, Nami Goto, Maki Hosoki, Yoshizo Matsuka

**Affiliations:** ^1^ Department of Stomatognathic Function and Occlusal Reconstruction Graduate School of Biomedical Sciences Tokushima University Tokushima Japan

**Keywords:** case study, metal allergy, patch test, titanium implant

## Abstract

Patch testing with metal reagents was positive on female patient with history of metal hypersensitivity after dental treatment. All of the dental restorations with metal components were removed, and subsequent oral rehabilitation utilizing dental implants and metal‐free prostheses was carried out. The treatments alleviate the presenting signs and symptoms.

## 1 INTRODUCTION

With the rapid development of technology and material science, more nonmetal materials are used in dental clinical treatment. However, we still need to use metal materials for the treatment. To increase the stability and corrosion resistance of materials, most of the metals are used in the form of alloys.^1^ The oral cavity has a complex environment; metal materials exist in the mouth for a long time. Daily life's acidic diet, food residue decomposition, and bacterial metabolism, such as acid production, reduce the pH value of the oral environment resulting in the chemical corrosion of the metal restorations.^2^

These metal ions may precipitate and are distributed throughout the body or absorbed by the local tissue. In vitro experiments also confirmed that nickel‐chromium alloy metal in the acidic artificial saliva environment would cause electrochemical corrosion and precipitation of the metal ions.^3^ It is not only possible to detect elevated metal ions in saliva, but some studies have shown that elevated levels of nickel and chromium are also detected in urine and blood of patients with nickel‐chromium alloy restorations.^4^, ^5^ It will lead to adverse reactions such as hypersensitivity or tissue damage. Metal allergic reactions are manifested not only in the oral mucosa but also in the extremities, limbs, and even on the skin. The clinical symptoms may not only be the subjective feelings such as the burning of oral mucosa, but also some objective damage such as moss‐like reaction, stomatitis, lip inflammation, and implant nail loosening may also occur. On the skin surface, the allergic manifestations may be in the form of eczema, contact dermatitis, plantar palm rash, etc.^6^, ^7^

According to our study of 212 suspected patients with metal allergy, the result of patch test showed the highest positive reaction toward Nickel (Ni) 25%, followed by Palladium (Pd) 24.4%, Chromium (Cr) 16.7%, Cobalt (Co) 15.9%.^8^ Also, Frigerio et al^9^ who performed patch tests on 100 patients who underwent first joint arthroplasty showed that the allergic rate was positive toward Ni 21%, Co 8%, Pd 3%, and Co–Pd was often accompanied by metal‐nickel cross‐allergy. Thyssen et al^10^ published on the analysis of contact allergic literature found that Ni allergy rate of 8.6% (0.7%‐27.8%) in North America and Western Europe is one of the most susceptible components causing contact allergy.

A case of a 54‐year‐old female patient with an allergy to metal jewelry is reported here. She presented with the allergic symptoms after the dental treatment. Clinical manifestations were mainly hyperemia in the oral mucosa and hand‐foot eczema. The results of the patch test showed that the patient had a positive reaction to a plurality of metallic metals mainly based on nickel and we treated by Ti implant dentures.

## 2 MATERIALS AND METHODS

### 2.1 History of the patient

A Japanese female patient had a history of rash in both of her feet when she was 45 years old. Her symptoms aggravated over the period to involve her hands as well until she was 51 years old. The dermatologist prescribed skin ointment, which did not improve her skin condition. She consulted a metal allergy expert and it was revealed that her dental treatment and skin allergy symptoms coincided.

Other significant medical history was that of cerebral infarction when she was 51 years old and the patient was taking oral aspirin for its prevention. She also gave a history of skin rashes due to cosmetics, earrings and necklace and a habit of smoking for 25 years, which she gave up.

### 2.2 Condition at the first visit

**Figure 1:**
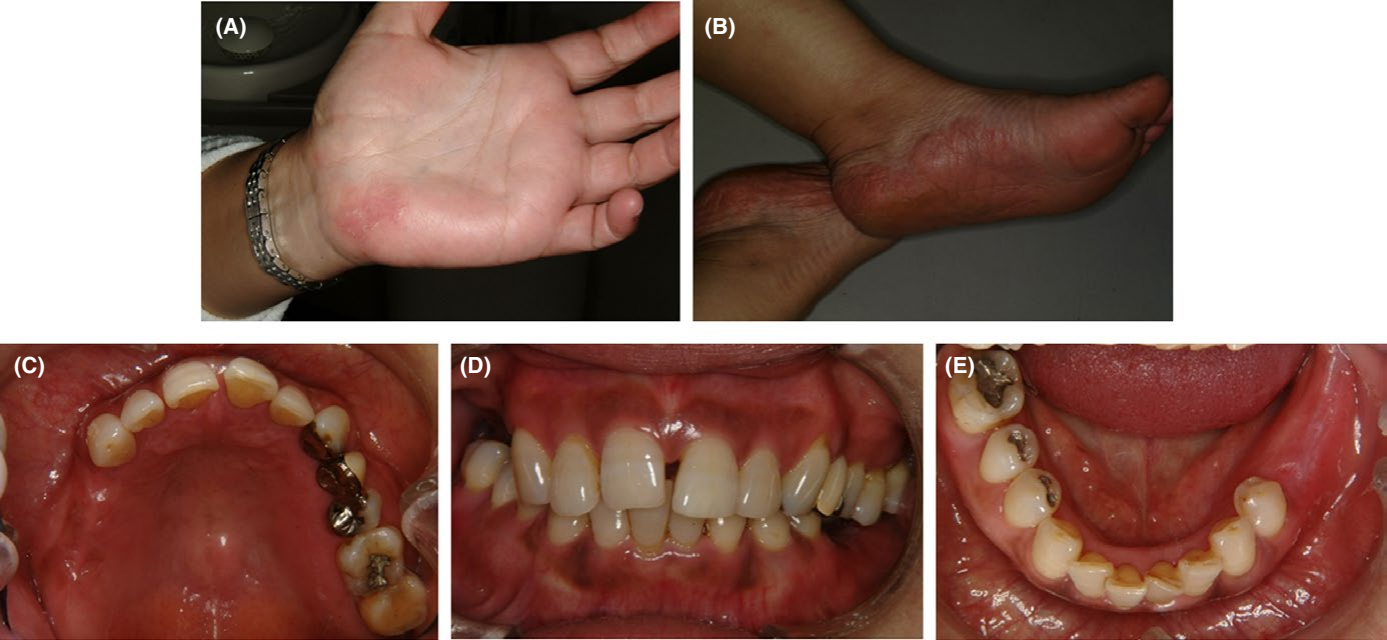
Clinical manifestation at the first dental appointment. A physical examination revealed red rashes on the palmoplantar surfaces of her hands (A) and feet (B). Intraoral examination findings were hyperemia of the oral mucosa (C‐E). There are some missing teeth

A physical examination revealed red rashes on the palmoplantar surfaces of her hands and feet (Figure 1A,B). Intraoral examination findings were hyperemia of the oral mucosa. Missing teeth were: maxillary right molars, mandibular left molars, and mandibular right second molar. Amalgam restorations were present on the maxillary left first molar and mandibular right first and second premolars and first molar. Metal 3‐unit fixed dental prostheses were present to replace missing left first premolar using canine and second premolar as abutment (Figure 1C‐E).

## 3 RESULTS

### 3.1 Skin patch test

The results of the skin patch test (Torii Pharmaceutical Corporation, Tokyo, Japan) for dental material in the dermatology department for general allergens showed a high positive reaction to Ni, Pd and molybdenum (Mo). The presence of these materials in the dental restorations was established by applying silica gel point technology to extract metal dust and X‐ray fluorescence (XRF) spectrometer to determine the metal constituent. It was confirmed that the Ni was present in the restorative material and was the cause of her presenting signs and symptoms.

### 3.2 Treatment for allergy

**Figure 2:**
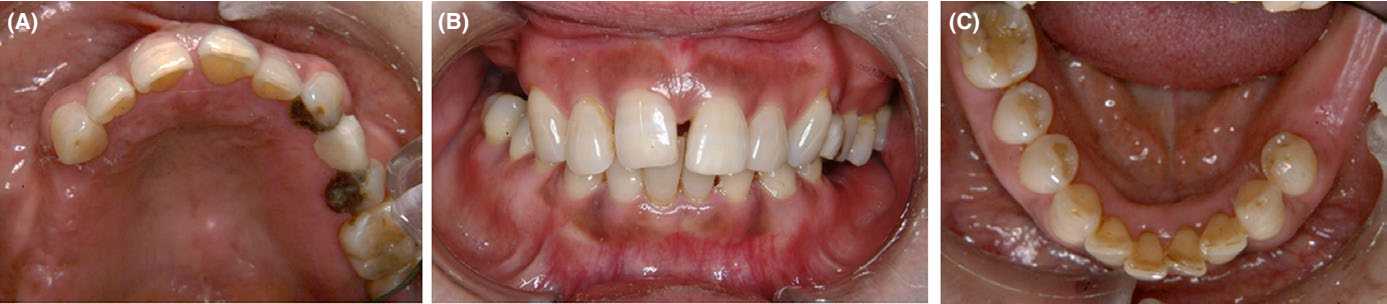
Clinical manifestation after the removal of dental prostheses. The all‐metal dental restorations were removed and replaced with composite restorations (A‐C)

All the metal dental restorations were removed and replaced with composite restorations; temporary bridge fabricated using hybrid composite resin to replace metal fixed dental prostheses. The progress of the case was monitored and allergic symptoms on hands and feet improved gradually (Figure 2).

### 3.3 Treatment for oral rehabilitation

**Figure 3:**
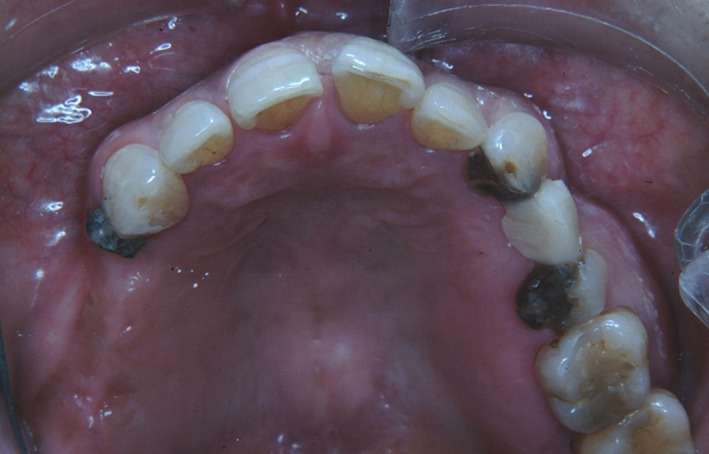
Titanium metal insert to check the allergic reaction. The small piece of Ti was cemented to the distal aspect of the maxillary right canine and observed for 6 mo

The two‐treatment options to restore oral function were metal‐free removable partial denture and implant‐supported fixed dental prostheses. As the patient was unable to use the removable dentures effectively, the implant‐supported prostheses to replace the missing teeth was the only treatment alternative. Although titanium (Ti) is a biocompatible metal, since the patient had a history of allergy to dental materials, before implantation a preventive test to check the patient's response to the Ti was carried out. The small piece of Ti was cemented to the distal aspect of the maxillary right canine and observed for 6 months^11^ (Figure 3). There was no allergic response (mucosal as well as cutaneous) detected and the previous allergic symptoms in hand and feet were not aggravated. An implant‐supported fixed dental prosthesis was planned at this stage. After a medical consultation, aspirin was stopped a week before implant surgery.

**Figure 4:**
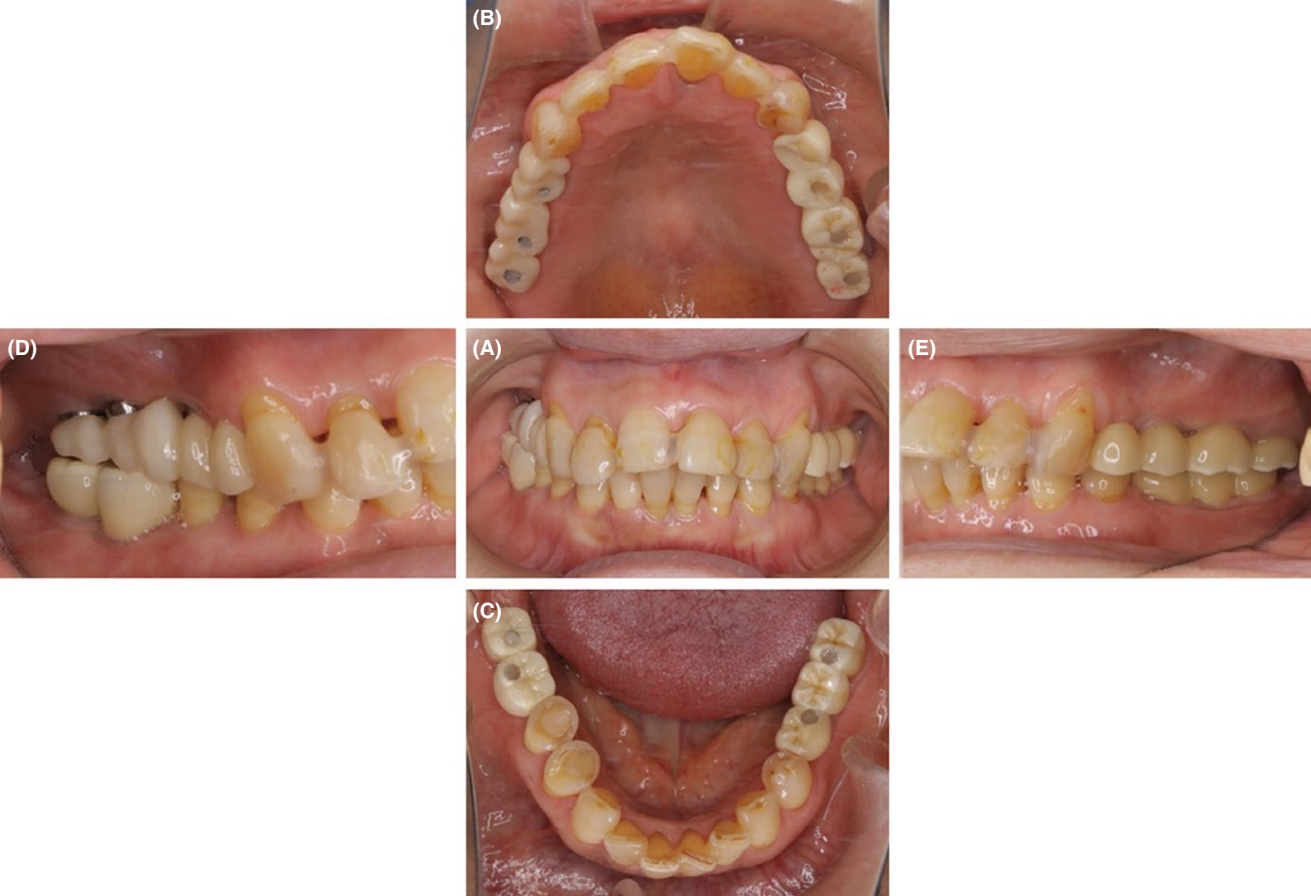
Full mouth rehabilitation of patient using dental implants and metal‐free prostheses. The implant dentures were restored with provisional restorations. Patient reported improvement in her oral function after the completion of oral rehabilitation

A two‐stage implant placement protocol was undertaken. Firstly, the mandibular implants were placed. After healing, the maxillary implants were placed for the missing teeth. After a healing period of 4 months for mandibular implants and 6 months for maxillary implants, a second‐stage surgery was performed for both the sites. Since the patient was not wearing any dental prostheses for her missing teeth, the natural teeth in the opposing arch (maxillary left first and second molars and mandibular right first and second premolars and first molar) were supra erupted. Therefore, to maintain the occlusal plane an intentional root canal treatment of the supra erupted teeth, followed by occlusal plane correction and composite resin restoration of the occlusal surface, was carried out. To achieve optimum crown length, surgical crown lengthening of the supra erupted teeth was performed in the periodontics department. The implant dentures were restored with provisional restorations. The patient reported improvement in her oral function after the completion of oral rehabilitation when she was 54 years old (Figure 4).

### 3.4 Relapse of allergic symptoms

After a period of 4 years, the patient reported a mild relapse of her allergic symptoms in her hands. Since she has multiple implants, she was worried that her relapse is because of her implants. To diagnose whether her symptoms were because of Ti implants, patch test for allergy to dental materials was carried out. We use 17 patch‐test metal reagents (Patch Test Reagents; Torii Pharmaceutical Corporation) and 11 custom‐made reagents. The reagents include metal elements that are mainly used for dental treatment in Japan. To rule out a false‐negative result, observation period for the patch test was extended from usual 48‐72 hours to 14 days with a regular observation on the 2nd, 3rd, 7th and 14th day. A positive reaction toward Ni, Pd, and Mo (similar to the previous results) and questionable reaction to tin (Sn), gold (Au) and zinc (Zi) was observed. Nevertheless, the allergic response to Ti was negative, consistent with the previous results. Patient's condition improved without any intervention.

## 4 DISCUSSION

Here, we report a patient with a history of allergy to metal ornaments and skin allergies after dental treatment. The patch test is the gold standard for diagnosing type IV hypersensitivity reaction.^12^ The initial skin patch test showed that the patient only had a strong positive reaction to Ni, but the negative reaction to Ti was also detected. When we removed the original metal prosthesis in the mouth, the patient's condition gradually improved and the further rehabilitation with implant‐supported denture was completed after the Ti clip was tested intraorally without the abnormal reaction. The postoperative recovery was also good, but after 4 years, there was very mild relapse of the symptoms in the area where the patient had allergies in the past. To confirm that the allergic symptoms were not because of implant treatment, patch test was repeated again, and the results were consistent with the earlier results. An allergy to Ti has not been detected on the second patch test also.

Although Ti is widely used in clinical practice because of its high biocompatibility, more and more literature in the recent past related to its sensitization and allergic response was reported.^13^ A case of contact dermatitis caused by the fractured end of the titanium nail fixation and the implant repair was reported.^14^ The investigators of Ti contact allergy patients found the allergy rate of 0.6%.^15^ In general, titanium is a material with high biocompatibility, and it is still the preferred material in clinical application. On the contrary, nickel and palladium are recognized as common metal allergens in life. At the same time, researchers have confirmed by the patch test, that women's positive reaction to palladium and nickel was significantly higher than men, speculated that the reason may be related to female wearing jewelry more than male, and the materials often contain elements such as palladium, nickel and other elements.^16^ In the present case, the patient had a history of allergy to metal jewelry, at the same time patch test results showed that the patients had a strong positive reaction to Ni, Pd, and other metals. In the present case, the relapse of the symptoms may be because of exposure to some unknown noxious allergen. No accurate diagnosis can be made at present and further observation of the patient's condition is recommended.

## 5 CONCLUSION

**Figure 5:**
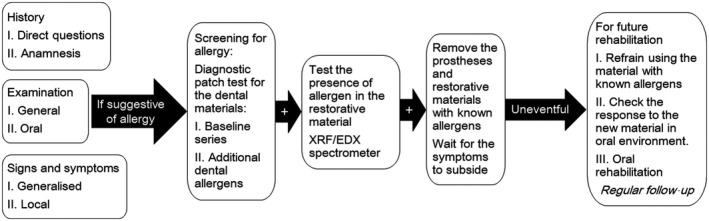
Protocol for diagnosing and managing patient with dental metal allergy

Symptomatic treatment and removal of materials‐causing allergy are the primary steps in managing a patient with known allergy to dental materials. To prevent relapse of the symptoms, it is important to ensure avoidance of exposure to the irritating substance. The dental material used for oral rehabilitation is in contact with the patient's body for a very long time. Screening for the allergy in the susceptible patients is highly recommended, especially with the history of allergy to day‐to‐day products such as cosmetics and jewelry. Any new material, even if it is Ti that is considered highly biocompatible and inert, for future rehabilitation should be pretested by patch test or by inserting the material directly in the oral cavity or both is recommended (Figure 5).

## CONFLICT OF INTEREST

None declared.

## AUTHORSHIP

HY: involved in patient care, made figure and tables, and participated in writing the manuscript. SA, JD, and NG: involved in patient care and discussion. MH: involved in metal allergy test. YM: involved in patient care and intellectual input.
